# Association between S100B Levels and Long-Term Outcome after Aneurysmal Subarachnoid Hemorrhage: Systematic Review and Pooled Analysis

**DOI:** 10.1371/journal.pone.0151853

**Published:** 2016-03-23

**Authors:** Pui Man Rosalind Lai, Rose Du

**Affiliations:** Department of Neurosurgery, Brigham and Women’s Hospital and Harvard Medical School, Boston, Massachusetts, United States of America; St Michael's Hospital, University of Toronto, CANADA

## Abstract

S100 calcium binding protein B (S100B), a well-studied marker for neurologic injury, has been suggested as a candidate for predicting outcome after subarachnoid hemorrhage. We performed a pooled analysis summarizing the associations between S100B protein in serum and cerebrospinal fluid (CSF) with radiographic vasospasm, delayed ischemic neurologic deficit (DIND), delayed cerebral infarction, and Glasgow Outcome Scale (GOS) outcome. A literature search using PubMed, the Cochrane Library, and the EMBASE databases was performed to identify relevant studies published up to May 2015. The weighted Stouffer’s Z method was used to perform a pooled analysis of outcome measures with greater than three studies. A total of 13 studies were included in this review. Higher serum S100B level was found to be associated with cerebral infarction as diagnosed by CT (*p*_*adj*_ = 3.1 x 10^−4^) and worse GOS outcome (*p*_*adj*_ = 5.5 x 10^−11^). There was no association found between serum and CSF S100B with radiographic vasospasm or DIND. S100B is a potential prognostic marker for aSAH outcome.

## Introduction

Subarachnoid hemorrhage (SAH) secondary to the rupture of an intracranial aneurysm remains a major cause of morbidity and mortality[1]. While aneurysmal rupture and recurrent hemorrhage contribute to a high initial mortality rate, delayed neurologic deterioration from ischemic complications continues to be a significant contributor to patient disability[2].

Cerebral vasospasm has largely accounted for a patient’s clinical deterioration and has been the major focus of clinical research. Radiographic evidence of vasospasm is observed in up to 67% of patients after aneurysmal SAH (aSAH)[3, 4]. Vasospasm as seen on an arteriogram may begin as soon as 2–3 days after SAH, peaking at 7–10 days and lasting up to 2 weeks[5]. While there is a high prevalence of radiographic vasospasm, only 20–30% of patients with vasospasm develop clinical deteriorations[3, 4, 6]. Recent trials have also failed to improve outcome from the reversal of vasospasm, suggesting that the pathophysiology may be more complex than radiographic vasospasm alone[7]. Thus, a new term has been introduced to describe the clinical decline, delayed cerebral ischemia, which is defined as focal neurological or cognitive deficits (delayed ischemic neurologic deficit, DIND), and/or the appearance of a new infarction on computed tomography (CT) or magnetic resonance (MR) imaging attributable to cerebral vasospasm (delayed cerebral infarction).

Various mechanisms have been proposed to identify the pathophysiologic mechanism of delayed cerebral ischemia. Early brain injury is a newly proposed concept which postulates that poor outcome after aSAH is a result of global ischemic injury to the brain[8, 9]. After the initial SAH, early brain injury involving inflammation, blood brain barrier disruption and increased intracranial pressure result in global ischemic injury and subsequent neurologic decline. As more research points to other mechanisms as the focus in delayed cerebral ischemia, prognostic and therapeutic targets have been shifted to these new directions.

Protein S100B is a calcium-binding protein expressed predominantly in astroglial cells and has been a well-studied marker for traumatic and ischemic injury. Serum S100B has also emerged as a potential candidate marker of the disruption of the blood brain barrier[10]. Given the relationship between global ischemic injury and clinical deterioration, it has been proposed that the same relationship may exist with S100B level and aSAH outcome[11, 12]. In the past decade, studies have attempted to investigate the association of S100B level in aSAH with the development of radiographic or clinical vasospasm[11, 12]. Meta-analyses have been useful tools in integrating findings from multiple studies to demonstrate genetic and molecular associations with cerebral vasospasm and SAH outcome. [13, 14] The goal of this study is to summarize the findings and perform a comprehensive review and pooled analysis that examine the associations between serum and cerebrospinal fluid (CSF) S100B with long-term patient outcome, radiographic vasospasm, DIND and delayed cerebral infarction.

## Methods

The following databases were used in a literature search for S100B with cerebral vasospasm, delayed cerebral ischemia or SAH outcome in humans published prior to May 2015: PubMed, the Cochrane Library, and EMBASE. Medical subject heading (MeSH) terms were used to identify studies containing the following subject terms: (“S100B” or “serum S100B” or “S100 calcium-binding protein beta subunit”) and (“cerebral vasospasm” or “intracranial vasospasm” or “delayed cerebral ischemia” or “DCI” or “delayed ischemic neurological deficits” or “DIND” or “delayed cerebral infarction”) and (“SAH” or “subarachnoid hemorrhage” or “aneurysmal subarachnoid hemorrhage” or “aneurysm”). The references of articles were also manually checked to search for additional studies.

The inclusion criteria were as follows: 1) retrospective or prospective cohort design, 2) statistical analysis with reporting of raw data, odds ratios with 95% confidence intervals or *p* values. Studies were excluded if they were identified as non-human studies, abstracts, case reports, commentaries, editorials or manuscripts unrelated to the research topic. Vasospasm or SAH not clearly due to a ruptured aneurysm were also excluded. If multiple measurements of S100B levels were independently reported in the same study, for example on day 0 and day 7 following ictus, the earliest timeframe measured was selected for the analysis.

Four parameters were assessed as outcome measures: radiographic vasospasm, DIND, delayed cerebral infarction, and Glasgow Outcome Scale (GOS) outcome. Each outcome measure was analyzed for both CSF and serum S100B independently. Radiographic vasospasm was defined as the narrowing of cerebral vessels visualized on diagnostic cerebral angiogram (DSA), computed tomography angiogram (CTA), or flow studies on Transcranial Doppler (TCD). DIND was defined as new onset of focal neurological deficit or deterioration in level of consciousness with no known intracerebral hemorrhage, rebleeding, or hydrocephalus, with or without radiographic evidence of vasospasm. Studies which defined vasospasm as clinical deterioration in addition to radiographic vasospasm were included under both radiographic vasospasm and DIND. Delayed cerebral infarction was classified as any new hypodensity in a vascular distribution on CT or MR imaging not seen on initial CT that is attributed to delayed cerebral ischemia. Patient outcome was defined as favorable (GOS 4–5) or unfavorable (GOS 1–3) from discharge to one-year followup. If GOS was measured at various time points within one study, values from the last followup was used in our analysis.

The following data were extracted from each study by both authors: author, year of publication, study size, sex and mean age of subjects, serum and CSF S100B levels, and the four outcome measures (radiographic vasospasm, DIND, delayed cerebral infarction, and Glasgow Outcome Scale (GOS).

Statistical analysis was performed using Review Manager (RevMan) version 5.3. The most commonly reported statistics were unpaired two-tailed or Mann-Whitney U tests with *p* values. A *p* value of 1 was assigned when studies reported non-significant findings with no exact *p* values available. Studies were excluded if no statistical method was described. All extracted *p* values were converted to one-tailed *p* values, and Stouffer’s Z method was used to perform pooled analyses for all 4 outcome measures. The weighted Stouffer’s Z method, or the Liptak-Stouffer test, is a validated tool in combining *p* values from independent tests and uses the square root of the sample size for the weighting [15]. A *p* value of 0.99 was used instead of p = 1 to avoid generating an infinite Z-score in the Stouffer’s test. The Benjamini and Hochberg correction was used to account for multiple testing. *P* < 0.05 was considered significant.

## Results

**Fig 1:**
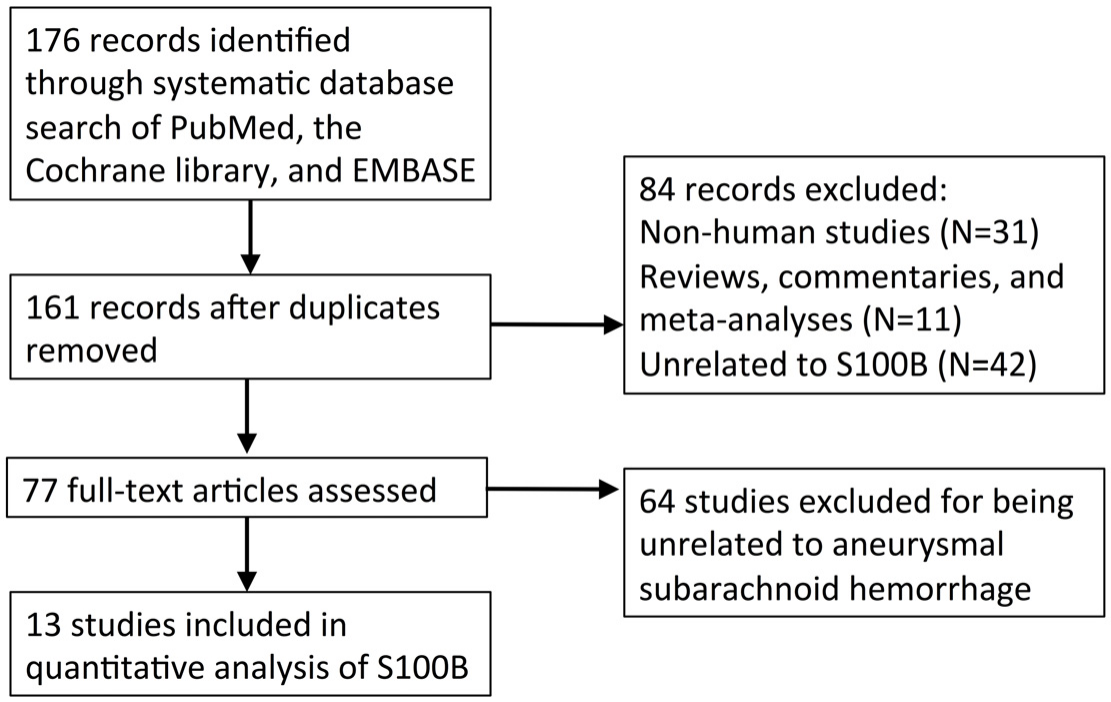
Literature search strategy.

A total of 161 studies were identified after removal of duplicate records (N = 15). Seventy-seven papers were identified for full-text assessment after the removal of non-human studies (*n* = 31), reviews, commentaries and meta-analyses (*n* = 11), and those studies that were unrelated to S100B (*n* = 42) (Fig 1). An additional 64 studies were excluded that were unrelated to aneurysmal subarachnoid hemorrhage, resulting in a final set of 13 records. The years of the studies included in this analysis ranged from 1997 to 2014.

The quality assessment of studies included in the pooled analyses is presented in S1 Table. Quality of each study was assessed based on external validity, internal validity, methods and statistical approach.

Study characteristics are detailed in Table 1. The thirteen studies included only cases of aSAH. All studies were written in English. A total of 575 patients were included with a mean age of 54.4 and 64% were female. Interventions in studies included surgical clipping alone, endovascular coiling alone and both. Two studies did not describe therapeutic procedures for aneurysms.

**Table 1:** Basic characteristics of the 13 studies included in the systematic review.

Source	N	Mean Age	Female (%)	Intervention: surgical clipping (%) or endovascular coiling (%)
Amiri 2013[11]	18	50	11 (61)	Clipping (19%) and coiling (81%)
Bellapart 2014[16]	20	58	15 (75)	Clipping and coiling
Kaneda 2010[12]	32	59	25 (78)	Clipping (100%)
Moritz 2010[17]	55	54	39 (71)	Clipping (77%), coiling (3.6%) and no intervention (18%)
Jung 2013[18]	18	-	10 (56)	Clipping (72%) and coiling (28%)
Kay 2003[19]	19	56	13 (68)	-
Oertel 2006[20]	51	51	31 (61)	Clipping (75%) and coiling (20%)
Pereira 2007[21]	51	51	31 (61)	Coiling (100%)
Sanchez-Pena 2008[22]	109	49	74 (68)	Coiling (100%)
Schick 2003[23]	43	-	25 (58)	Clipping (100%)
Siman 2011[24]	14	54	10 (71)	Clipping and coiling
Weiss 2006[25]	74	58	42 (57)	Clipping (72%) and coiling (28%)
Wiesmann 1997[26]	71	55	42 (59)	-

A summary of the studies describing the method of S100B detection and outcome measures evaluated are described in Table 2. All CSF and serum samples were collected within 2 weeks following ictus. Eight studies evaluated radiographic vasospasm confirmed by CTA, DSA or TCD and three studies included clinical deterioration as the definition of symptomatic vasospasm or DIND. Delayed cerebral infarction by CT was detailed in 5 studies. A total of 10 studies used GOS as outcome measurements within 1 year after discharge.

**Table 2:** Summary of the literature on CSF and serum S100B as biomolecular markers for radiographic vasospasm, DIND, delayed cerebral infarction and GOS outcome after aneurysmal SAH.

Source	N	Detection substrate	Sample collection (days following ictus)	Radiographic vasospasm*	DIND	Delayed cerebral infarction	Long-term GOS Outcome
Amiri 2013[11]	18	CSF(ventricular)	Days 1–3	X (CTA)	X (clinical deterioration)	-	-
	18	Blood	Days 1–3	X (CTA)	X (clinical deterioration)	-	-
Bellapart 2014[16]	20	Blood (arterial)	Day 5	X (DSA or CTA)	-	-	-
Kaneda 2010[12]	32	CSF (ventricular or cisternal)	Day 3	-	-	-	↑ (at 6 months)
Kay 2003[19]	19	CSF (ventricular)	Within 3 days	-	-	-	X (at 3 months)
Moritz 2010[17]	55	CSF (ventricular)	Mean over days 0–8	X (TCD**)	-	↑ (CT)	↑ (at discharge)
	55	Blood (arterial)	Mean over days 0–8	X (TCD**)	-	↑ (CT)	↑ (at discharge)
Jung 2013[18]	18	Blood	Days 0–12	X (DSA)	-	↑(CT)	-
Oertel 2006[20]	51	Blood (venous, central line)	Days 0–3	↓ (clinical deterioration+TCD†)	↓ (clinical deterioration+TCD)	X (CT)	↑ (at 6 months)
Pereira 2007[21]	51	Blood (venous)	Mean over days 1–8	-	-	-	↑ (12 months)
Sanchez-Pena 2008[22]	109	Blood (venous)	Daily from days 1–15	-	-	-	↑ (12 months)
Schick 2003[23]	43	Blood (venous)	Daily from days 0–9	-	-	↑ (CT)	↑ (discharge and within 2y)
Siman 2011[24]	15	CSF (ventricular)	Mean over days 3–10	X (TCD††)	-	X (CT)	X (6–9 months)
Weiss 2006[25]	74	Blood (venous)	Day 1	X (Clinical deterioration+DSA)	X (clinical deterioration + DSA)	-	↑ (6 months)
Wiesmann 1997[26]	71	Blood	Day 1	-	-	-	↑ (6 months)

* ↑ (positive correlation); ↓ (negative correlation); X (no statistical significance);—(not evaluated)

** Vasospasm was defined as mean blood flow velocity >120 cm/s, or daily change in mean blood flow velocities >50cm/s

† Vasospasm was defined as mean blood flow velocity of the middle cerebral artery >120 cm/s and a Lindegaard ratio >3

†† Vasospasm was defined as mean blood flow velocity >125 cm/s in the anterior circulation or >100 cm/s in the posterior circulation and a Lindegaard ratio > 3

Pooled analysis was performed when there were more than three studies representing an outcome measure. The one-sided *p* value for each study was obtained (Table 3). Overall results for the pooled analyses are shown in Table 4. Each outcome measure is described in detail below.

**Table 3:** A total of 13 studies were included in this systematic review of S100B with radiographic vasospasm, DIND, delayed cerebral infarction, and GOS outcome. Reported two-tailed *p* values were converted to one-tailed *p* value to perform the Stouffer’s method for pooled analysis.

Source	Sample size	One-sided *p* value
**Radiographic vasospasm (serum)**		
Amiri 2013[11]	18	1
Bellapart 2014[16]	20	0.96
Jung 2013[18]	18	1
Moritz 2010[17]	55	1
Oertel 2006[20]	51	1
Weiss 2006[25]	74	1
**Radiographic vasospasm (CSF)**		
Amiri 2013[11]	18	1
Moritz 2010[17]	55	1
Siman 2011[24]	15	0.48
**Delayed ischemic neurologic deficits (serum)**		
Amiri 2013[11]	18	1
Oertel 2006[20]	51	1
Weiss 2006[25]	74	1
**Delayed cerebral infarction (serum)**		
Jung 2013[18]	18	0.06
Moritz 2010[17]	55	0.002
Oertel 2006[20]	51	0.18
Schick 2003[23]	43	0.02
**Delayed cerebral infarction (CSF)**		
Moritz 2010[17]	55	0.1
Siman 2011[24]	15	0.26
**Long-term GOS outcome (serum)**		
Moritz 2010[17]	55	0.0002
Oertel 2006[20]	51	0.002
Pereira 2007[21]	51	0.0002
Sanchez-Pena 2008[22]	109	0.0002
Schick 2003[23]	43	0.02
Weiss 2006[25]	74	0.06
Wiesmann 1997[26]	71	0.002
**Long-term GOS outcome (CSF)**		
Kaneda 2010[12]	32	0.1
Kay 2003[19]	19	1
Moritz 2010[17]	55	0.002
Siman 2011[24]	15	0.28

**Table 4:** Pooled analyses were performed for outcome measures with at least 3 studies represented.

Outcome measure	Total # of studies	Total N	Z score	*p* value (Stouffer)	*p* value adj.*
Radiographic vasospasm (serum)	6	236	-5.30	1.00	1.0
Radiographic vasospasm (CSF)	3	88	-2.87	1.00	1.0
DIND (serum)	3	143	-3.89	1.00	1.0
Delayed cerebral infarction (serum)	4	167	+3.71	1.04 x 10^−4^	3.1 x 10^−4^
GOS Outcome (serum)	7	454	+6.72	9.22 x 10^−12^	5.5 x 10^−11^
GOS Outcome (CSF)	4	121	+1.88	2.99x10^-2^	6.0 x 10^−2^

*Stouffer’s *p* values were adjusted using the Benjamini and Hochberg method for multiple testing.

Evidence of radiographic vasospasm by DSA, TCD or CTA and its association with serum and CSF S100B was evaluated in 6[11, 16–18, 20, 25] and 3[11, 17, 24] studies (Table 3), respectively. No association was found between serum or CSF S100B levels with radiographic vasospasm. Thus the pooled analysis also demonstrated no statistical significance (*p* = 1.0) (Table 4).

Three studies[11, 20, 25] evaluated the association between serum S100B level and neurologic deterioration attributed to ischemia (Table 3). Oertel *et al*. found lower levels of serum S100B to be associated with clinical deterioration[20], while the other two studies did not find this effect. The pooled analysis did not find an association between DIND with serum S100B (Table 4). There were no studies in our analysis that evaluated S100B in CSF with DIND.

Among the four studies[17, 18, 20, 23] examining the association between serum S100B with delayed cerebral infarction, three found higher levels to be associated with increased risk of infarction (Table 3). The pooled analysis summarized this finding with a significance at *p* = 1.0 x 10^−4^ (*p*_*adj*_ = 3.1 x 10^−4^**)**. Pooled analysis was not generated for delayed cerebral infarction with CSF S100B as only two studies[17, 24] were included in this outcome, although both did not demonstrate a significant association.

Seven studies[17, 20–23, 25, 26] met the criteria for evaluating serum S100B level with GOS outcome from the time of discharge up to one-year followup (Table 3). All studies reported higher serum S100B level with worse outcome. The pooled analysis summarized this association between serum S100B with worse clinical outcome (*p*_*adj*_ = 5.5 x 10^−11^). Four studies[12, 17, 19, 24] evaluated CSF S100B with clinical outcome. The pooled analysis suggests a trend towards higher S100B CSF level associating with worse GOS outcome but was not significant after multiple testing adjustment (*p* = 3.0x10^-2^, *p*_adj_ = 6.0 x 10^−2^).

## Discussion

The identification of biomolecular markers for the prediction of outcome after aSAH has been widely studied. S100B is a member of the thirteen S100 calcium-binding family proteins of the EF hand motif, the most common calcium-binding motif in proteins[27]. S100B is primarily expressed in the cytoplasm of astrocytes and has been implicated in the regulation of cell cycle progression and differentiation through microtubule and intermediate filament assembly[28]. The protein was initially described in 1965 by Moore *et al*.[29] and subsequently, the alteration of S100B gene has been associated with many neurological diseases including Alzheimer’s, amyotrophic lateral sclerosis and epilepsy[30–32]. More importantly, S100B has emerged as a marker of the acute phase of neurological damage. The protein is released by astrocytes following acute brain injury and can be detected in serum if the blood brain barrier is compromised. S100B has also been described as a prognostic marker in the prediction of outcome after traumatic brain injury and large volume cerebral infarction[33–35]. Furthermore, an elevated serum S100B level has been demonstrated in ischemic stroke and is associated with worse outcome after a stroke[36, 37].

Weismann *et al*.[26] were the first to investigate the association between outcome after aSAH with S100B. They were also the first to report the measurement of S100B in serum as a potential non-invasive method to predict disease. The first published report to examine the association between radiographic vasospasm and S100B was by Oertel *et al*.[20] in 2006, albeit finding no correlation. Subsequently, more studies have tried to reexamine and replicate these studies to clarify the relationship between S100B with SAH outcome and vasospasm. The goal of our review and pooled analysis is to summarize these findings.

In this review, a total of 13 studies with 575 patients were identified in our literature search to analyze the association between serum and CSF S100B with radiographic vasospasm, DIND, delayed cerebral infarction and GOS outcome. There was a high association between elevated serum S100B level and cerebral infarction as demonstrated on CT. However, the temporal relationship between S100B levels and the appearance of infarctions on CT was not evaluated and the infarctions that are reported in the studies are not clearly non-treatment related. Thus the association here may simply be the result of brain injury regardless of cause. Indeed, a comparable relationship between serum S100B and ischemic stroke has been described in the literature. Hermann *et al*. found that increased serum S100B was associated with irreversible ischemic stroke, but not with reversible lesions[38]. It has been postulated that S100B is a marker signaling irreversible ischemic damage, thus explaining its correlation with infarction and subsequent worse long-term prognosis. Similarly, serum S100B may be a marker for infarction seen on CT secondary to severe vasospasm, but is not associated with reversible radiographic vasospasm. Given the current hypotheses of the complex mechanisms contributing to delayed cerebral ischemia, there may be other pathways independent of vasospasm which explain the increased serum S100B level and its relationship with ischemia despite its lack of association with radiographic vasospasm.

In addition to cerebral infarction, there is a high association between elevated serum S100B level and worse long-term GOS outcome. This association between S100B level and GOS outcome is already apparent at discharge, as both Jung *et al*. and Schick *et al*. described[18, 23]. Our pooled analysis included GOS outcome studies from discharge up to 1-year follow up, resulting in a total of 7 studies included in the evaluation for serum S100B. Despite the small sample sizes in each study, all described a similar relationship and positive correlation. Thus, the pooled analysis yielded a strong association. Delayed cerebral infarction with or without symptoms has been found to be a major predictor of poor outcome. Thus, it is not surprising that GOS outcome has an association with S100B, which is correlated with infarction itself[39]. There was a trend towards association between CSF S100B level and outcome, although it was not statistically significant in our study.

A number of limitations should be considered in this study. Although the weighted Stouffer’s method is a validated statistical approach to combine studies, many studies represented in this review did not include sufficient statistical information to perform a more rigorous pooled analysis. We were thus unable to assess heterogeneity and perform a meta-analysis using the random effects or fixed effects model. Despite this limitation, all studies reported higher S100B levels with worse clinical outcome, suggesting these effects are evident even with small sample sizes in each study. In contrast, no study demonstrated an association of radiographic vasospasm with S100B levels, suggesting strong evidence for a lack of association. However, the results for radiographic vasospasm are limited by the different criteria used in different studies (TCD based vs CTA/DSA based). Nevertheless, it has been shown that TCDs are highly specific for radiographic vasospasm as detected by DSA[40]. S100B levels have been reported to be higher after surgical clipping than endovascular coiling[25], but it was not possible to perform a subgroup analysis given the small number of studies. Future research stratifying the method of treatment may be helpful in further understanding this relationship.

## Conclusions

Using a pooled analysis, we found higher serum S100B level to be associated with cerebral infarction and worse long-term outcome. However, we found no correlation with radiographic or clinical vasospasm. Further investigations of S100B may be helpful in validating the protein as a reliable predictor of long-term SAH outcome.

## Supporting Information

S1 File PRISMA checklist. (DOC)

S1 Table Quality assessment of studies included in the pooled analyses. (DOCX)
