# Supplementary material for: Association between S100B Levels and Long-Term Outcome after Aneurysmal Subarachnoid Hemorrhage: Systematic Review and Pooled Analysis
Source: PLoS One. 2016 Mar 23;11(3):e0151853. doi: 10.1371/journal.pone.0151853 (PMC4805236; doi:10.1371/journal.pone.0151853)
Supplement: S1 Table — (DOCX) [file pone.0151853.s002.docx]

**Supplementary table 1:** Quality assessment of vasospasm and SAH outcome studies on S100B. √ = yes, X = no, N/A = not enough information.

| First author and published year | External validity | | Internal validity/Methods | | | | | | Statistical validity | |
| --- | --- | --- | --- | --- | --- | --- | --- | --- | --- | --- |
|  | Prospective Design | Sample from aSAH only | Detailed eligibility criteria in addition to aSAH | Specified S100B analysis | Vasospasm/DCI Outcome defined | GOS Outcome defined | GOS follow-up rate >80% | Outcome blinded | Statistical Analyses for S100B described or raw values given | Sufficient descriptive data (e.g. age, sex, patient outcome) |
| Amiri 2013^11^ | X | √ | √ | √ | √ | N/A | N/A | N/A | √ | √ |
| Bellapart 2014^20^ | √ | √ | X | √ | √ | N/A | N/A | N/A | X | √ |
| Kaneda 2010^12^ | √ | √ | X | √ | N/A | √ | √ | √ | √ | √ |
| Moritz 2010^17^ | √ | √ | √ | √ | √ | √ | N/A | N/A | √ | √ |
| Jung 2013^18^ | X | √ | √ | √ | √ | N/A | N/A | N/A | √ | √ |
| Kay 2003^19^ | √ | √ | X | √ | N/A | √ | N/A | N/A | √ | N/A |
| Oertel 2006^20^ | √ | √ | √ | √ | √ | √ | √ | N/A | √ | √ |
| Pereira 2007^21^ | √ | √ | √ | √ | N/A | √ | √ | N/A | √ | √ |
| Sanchez-Pena 2008^22^ | √ | √ | √ | √ | N/A | √ | √ | N/A | √ | √ |
| Schick 2003^23^ | √ | √ | √ | √ | N/A | √ | N/A | N/A | √ | √ |
| Siman 2011^24^ | √ | √ | √ | √ | √ | √ | X | √ | √ | √ |
| Weiss 2006^25^ | √ | √ | √ | √ | √ | √ | N/A | √ | √ | √ |
| Wiesmannn 1997^26^ | √ | √ | √ | √ | N/A | √ | N/A | N/A | √ | √ |
